# Supplementary material for: The Transmembrane Protease Serine 2 (TMPRSS2) Non-Protease Domains Regulating Severe Acute Respiratory Syndrome Coronavirus 2 (SARS-CoV-2) Spike-Mediated Virus Entry
Source: Viruses. 2023 Oct 19;15(10):2124. doi: 10.3390/v15102124 (PMC10612036; doi:10.3390/v15102124)
Supplement: Supplementary file 1 [file viruses-15-02124-s001.zip › viruses-2668906-supplementary.v2.pdf]

Supplementary

The Transmembrane Protease Serine 2 (TMPRSS2) Non-Protease Domains Regulating Severe Acute Respiratory Syndrome Coronavirus 2 (SARS-CoV-2) Spike-Mediated Virus Entry

Romano Strobelt, Julia Adler and Yosef Shaul *

Department of Molecular Genetics, Weizmann Institute of Science, Rehovot 7610001, Israel
* Correspondence: yosef.shaul@weizmann.ac.il

Supplementary Figures

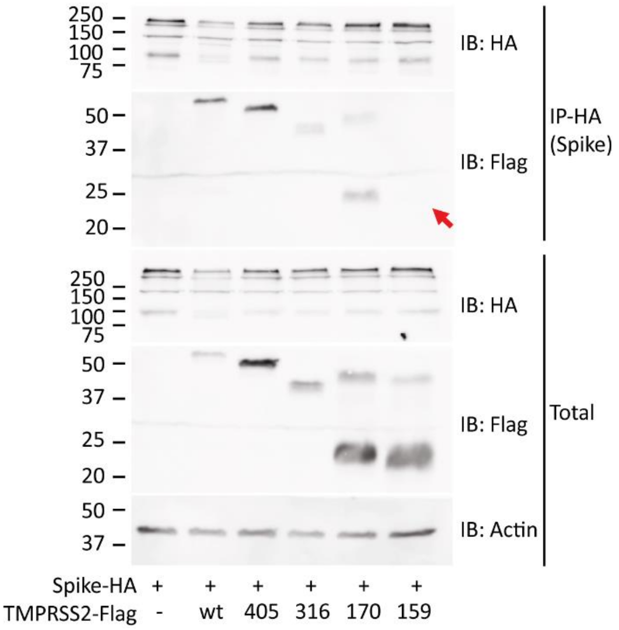

Figure S1: The reciprocal experiment from WB Figure 3b Mapping the Spike-TMPRSS2 interacting region. HEK293T were transfected with the indicated plasmids and IPed with HA-beads and WB were performed as above. Red arrow shows the expected location of the 1-159-TMPRSS2-Flag band within IP-Flag blot.

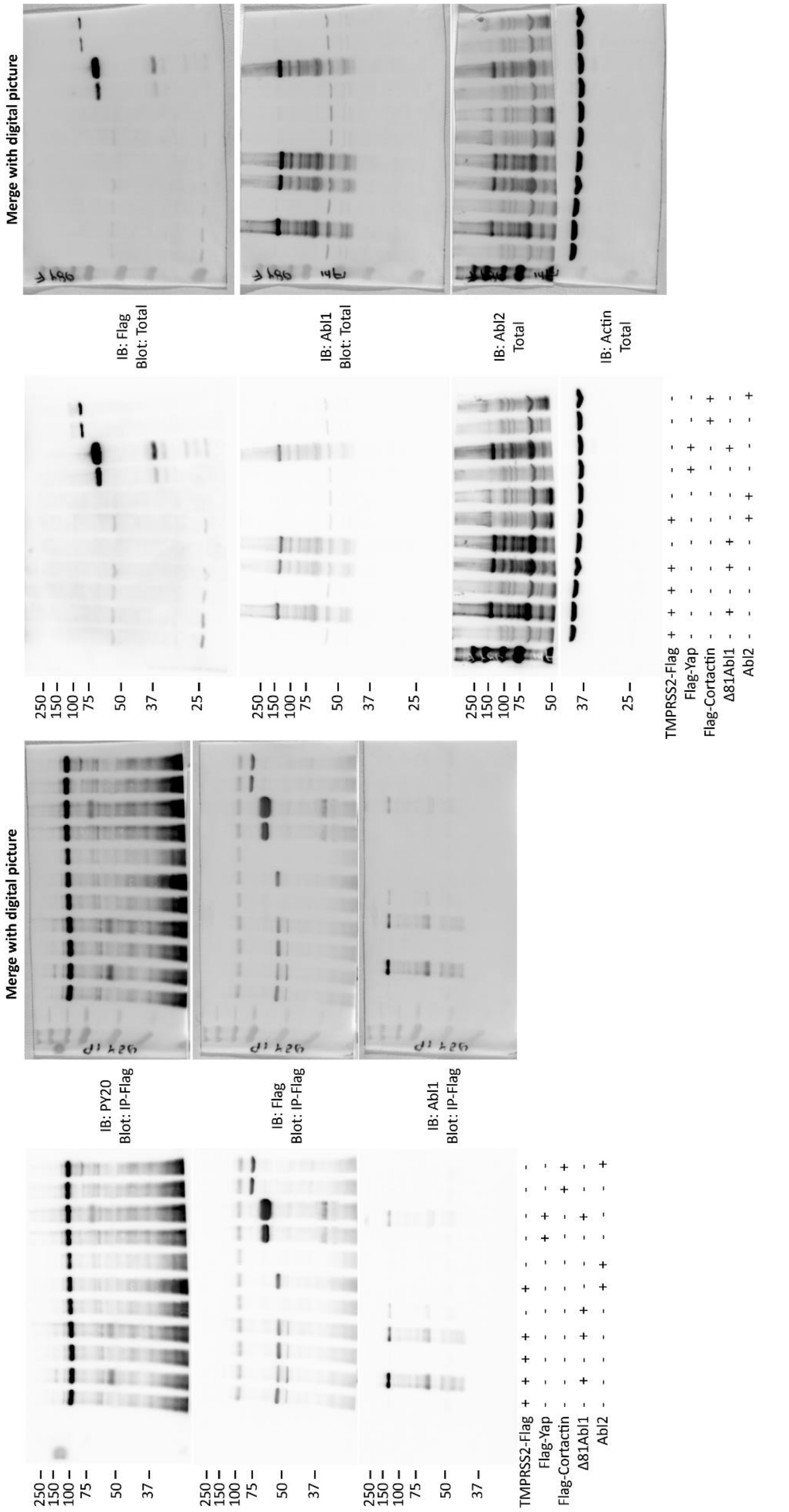

Figure S2: Original western blot image from Flag-IP Figure 1b. Blots were incubated and re-stained in following order for Flag-IP blot: 1) Anti-mouse-PY20, 2) Anti-mouse-Flag (TMPRSS2, Yap or Cortactin), 3) Anti-mouse-Abl1 and for the total blot: 1) Anti-mouse-Flag (TMPRSS2, Yap or Cortactin), 2) Anti-mouse-Abl1, 3) 2) Anti-mouse-Abl2 and 4) Anti-mouse-Actin

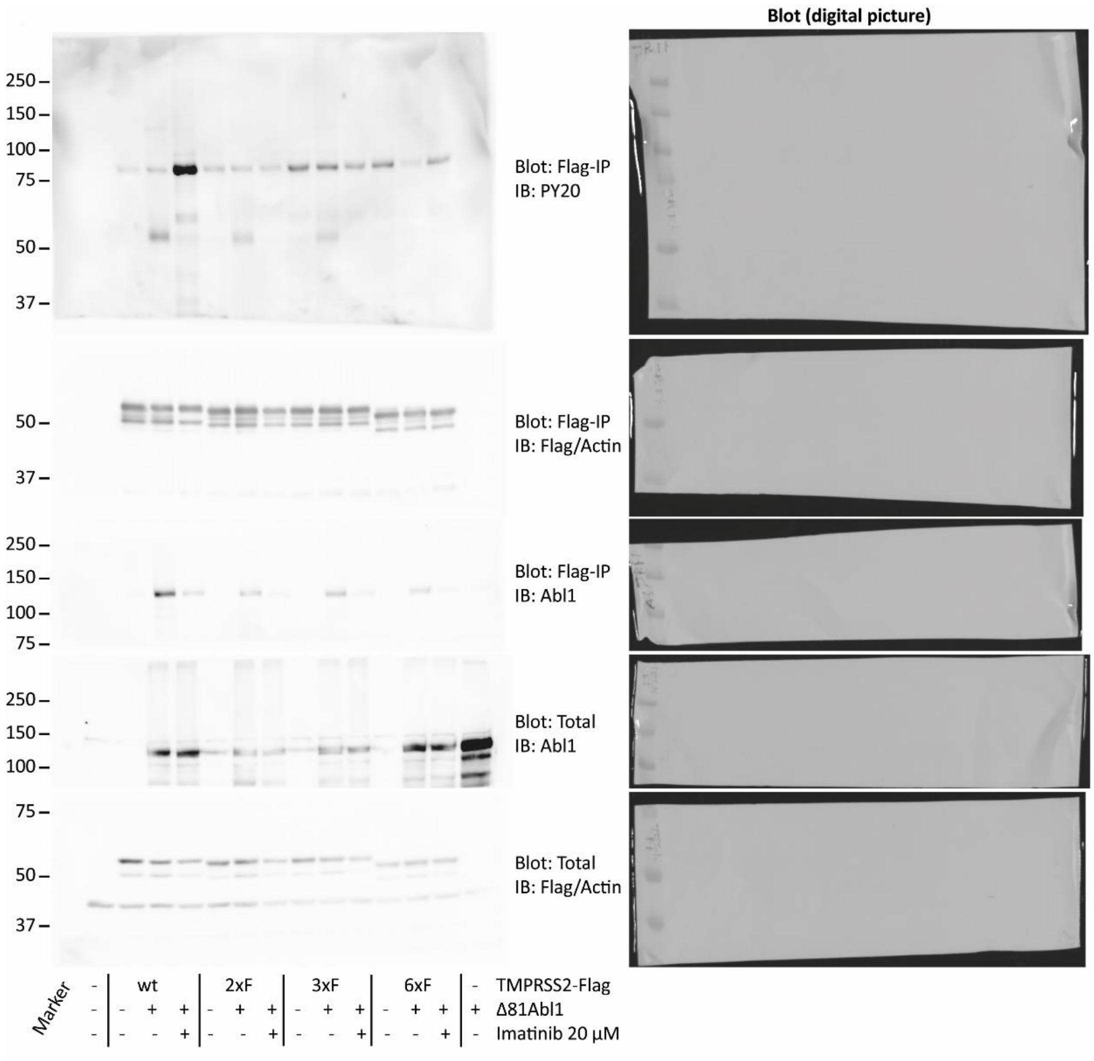

Figure S3: Original western blot image from Flag-IP Figure 1c. Blots were cut between lane 75 kDa and treated with indicated antibodies. The Flag-IP blot was cut after analysis of PY20 antibody treatment.

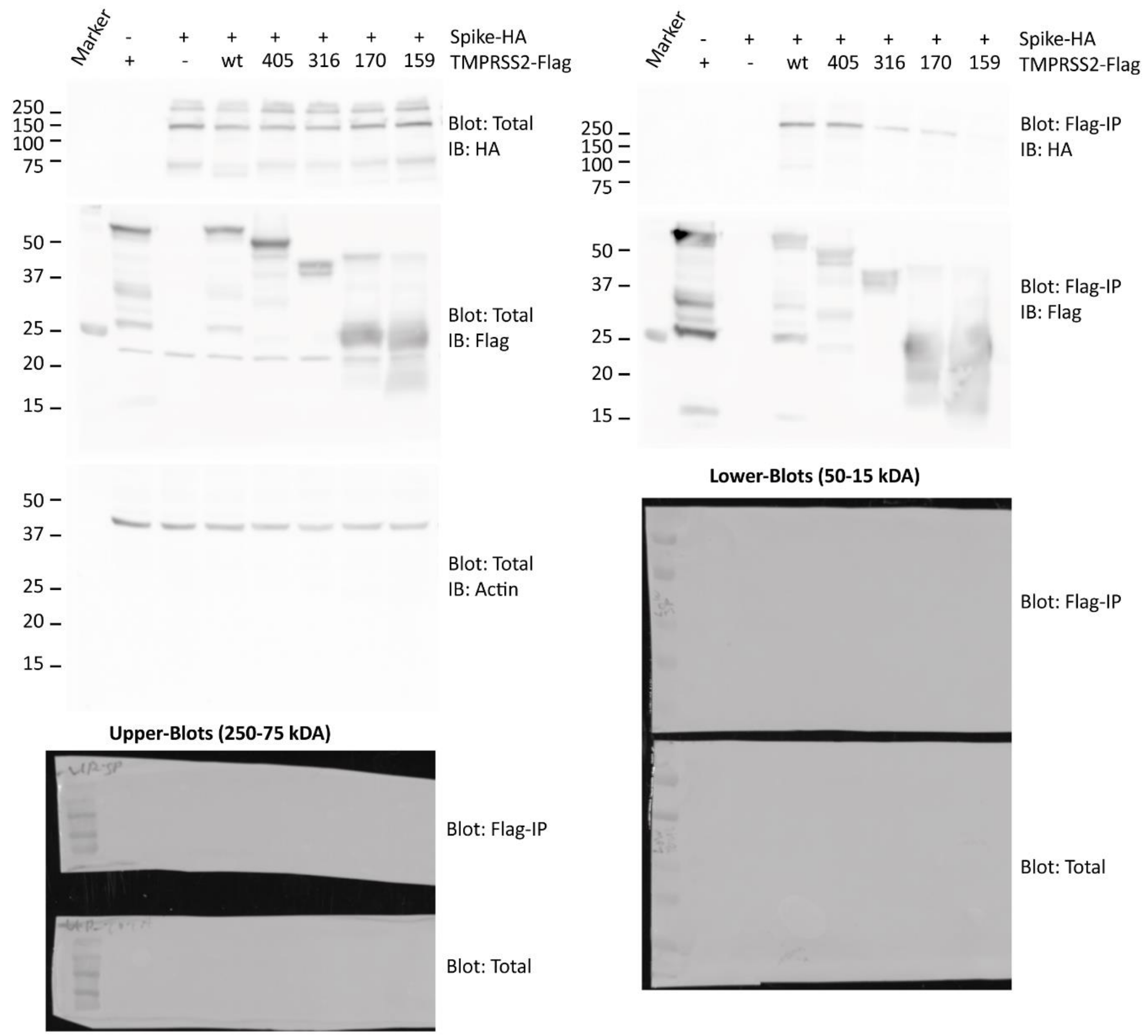

Figure S4: Original western blot image from Flag-IP Figure 3b. Blots were cut between lane 75 kDa and the upper blots were treated with Anti-mouse-HA (TMPRSS2) antibodies while lower blots was treated with Anti-rabbit-Flag (Spike) and two days later with Anti-mouse-Actin antibodies.

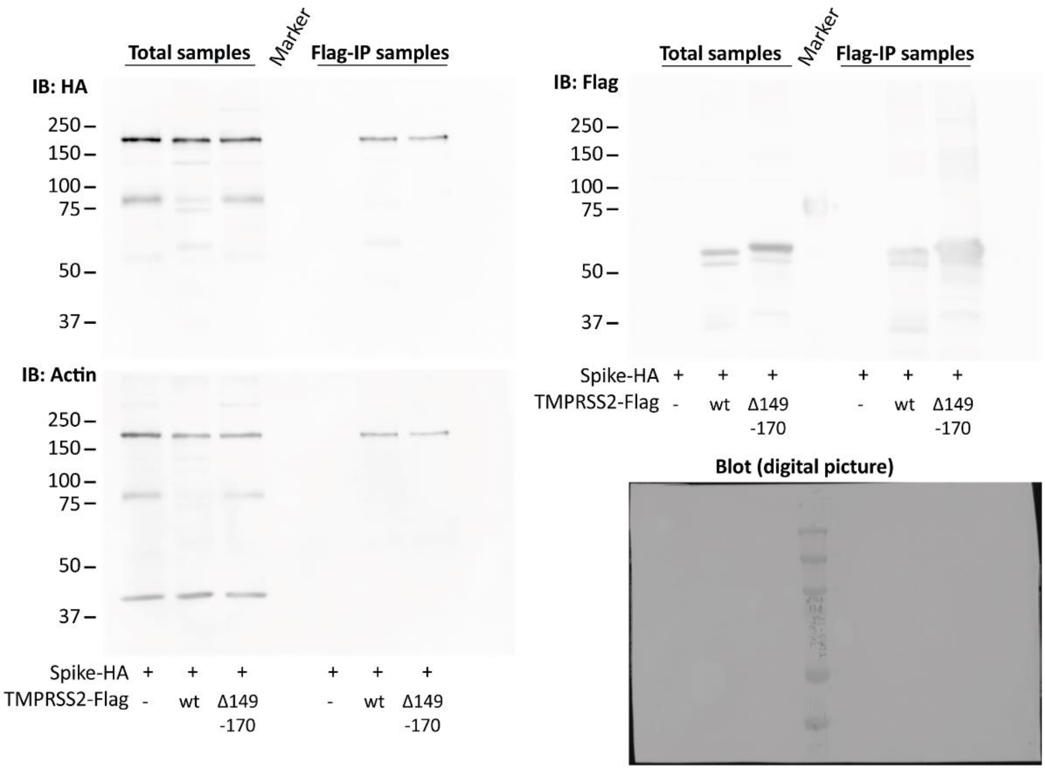

Figure S5: Original western blot image from Flag-IP Figure 3d. Blot was incubated and re-stained in following order: 1) Anti-mouse-HA (Spike), 2) Anti-rabbit-Flag (TMPRSS2) and 3) Anti-mouse-Actin.

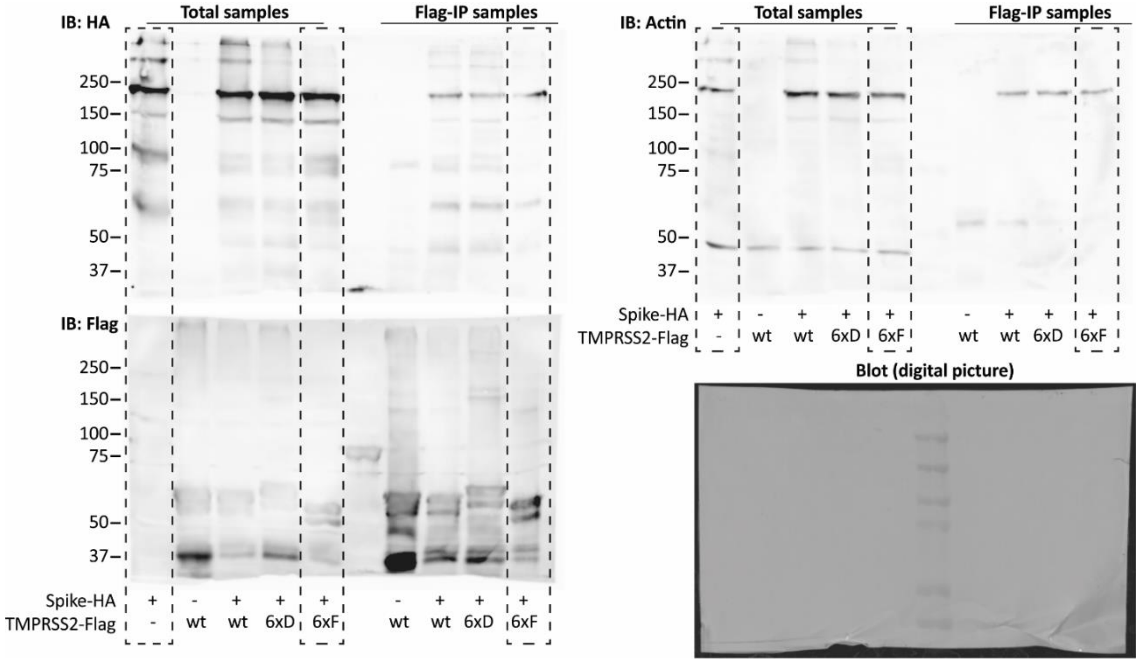

Figure S6: Original western blot image from Flag-IP Figure 4a. Dashed box shows excluded lanes from figure 4a in main text. Blots were incubated and re-stained in following order: 1) Anti-mouse-HA (Spike), 2) Anti-rabbit-Flag (TMPRSS2) and 3) Anti-mouse-Actin.

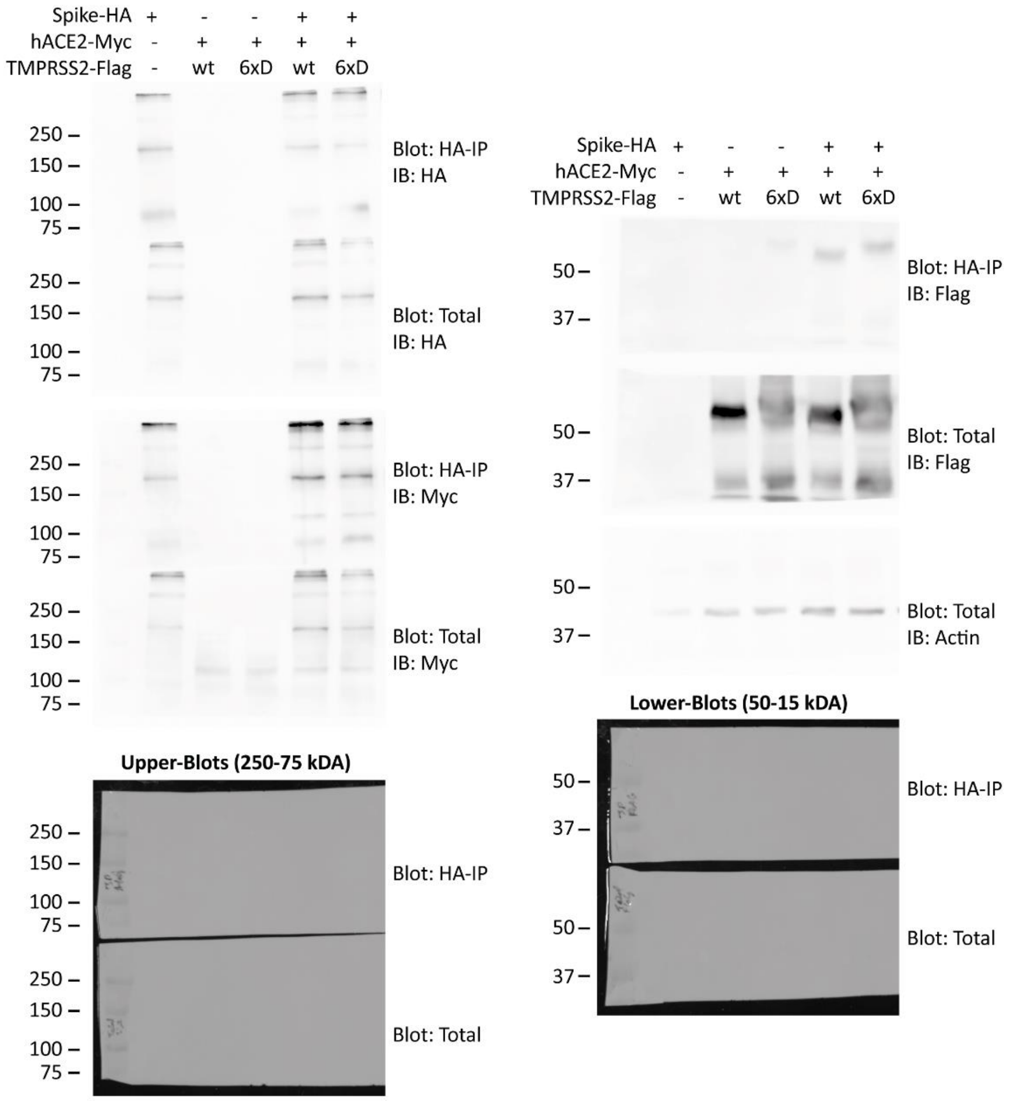

Figure S7: Original western blot image from Flag-IP Figure 4c. Blots were cut between lane 75 kDa and the upper blots were treated with Anti-mouse-HA (Spike) and anti-Myc (hACE2) antibodies while lower blots was treated with Anti-rabbit-Flag (TMPRSS2) and with Anti-mouse-Actin antibodies. Result was verified with an additional experiment.

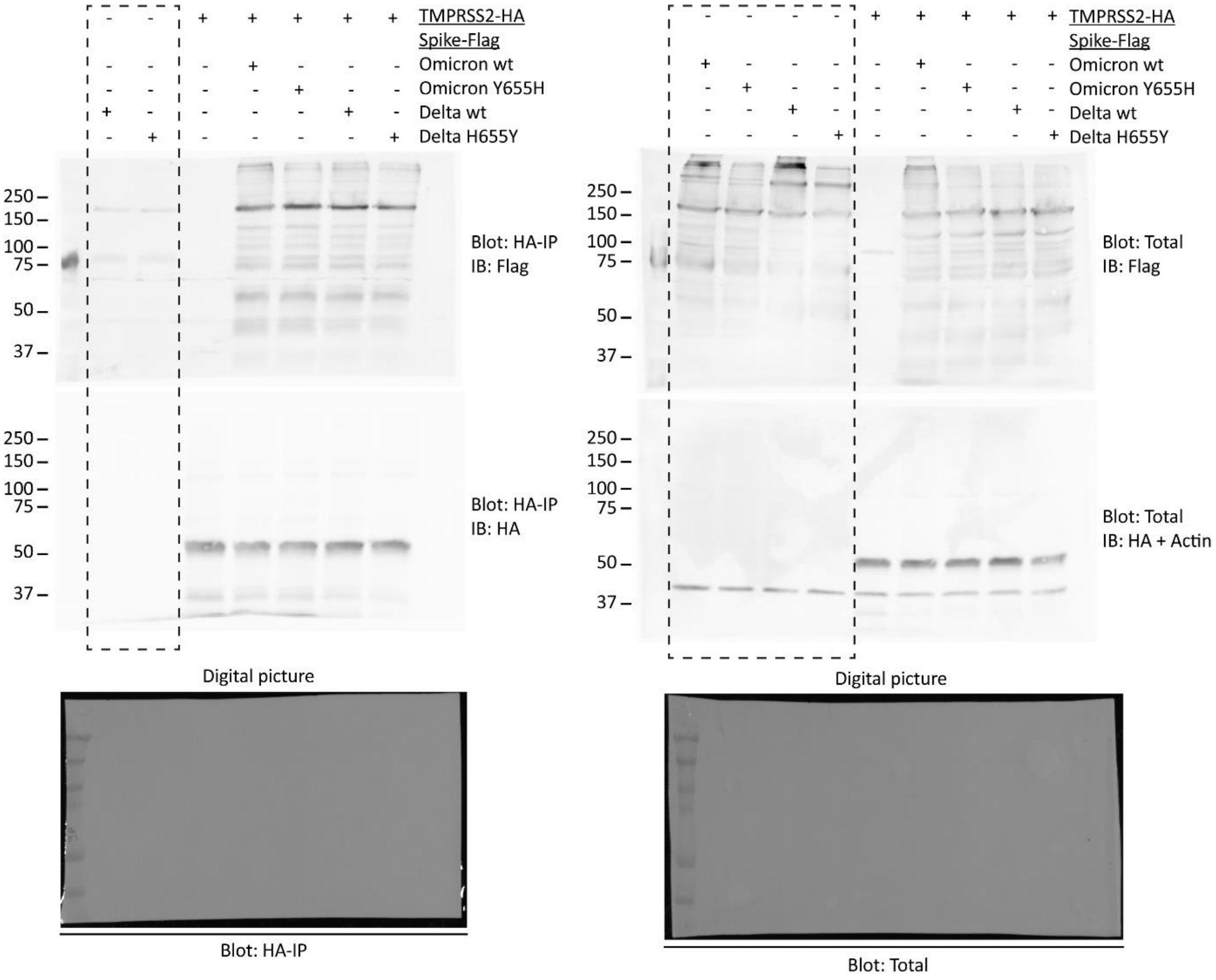

Figure S8: Original western blot image from Flag-IP Figure 5b. Dashed box shows excluded lanes from figure 5b in main text. Blots were incubated and re-stained in following order: 1) Anti-rabbit-Flag (Spike), 2) Anti-mouse-HA (TMPRSS2) + Anti-mouse-Actin.

Supplementary Table

Supplement Table S1: List of Primer used for cloning of described constructs.

Name	Sequence
**TMPRSS2_Fw_NheI**	AATTGCTAGCGGGCCCACCATGGCTTTGAACTCAGGGTCACC
**TMPRSS2_Rv_Flag_XbaI**	AATTTCTAGATTACTTGTCGTCATCGTCTTTGTAGTCGCCGTCTGC C CTCATTTGTCGATA
**TMPRSS2_Rv_HA-tag_XbaI**	AATTTCTAGATTAtgcataatccggaacatcatacggataGCCGTCTGCCCT CATTTGTCGATA
**TMPRSS2-1347_Rv_Flag_XbaI**	CCAATTTCTAGATTACTTGTCGTCATCGTCTTTGTAGTCCTTCGAA GT GACCAGAGG
**TMPRSS2-1215_Rv_Flag_XbaI**	CCAATTTCTAGATTACTTGTCGTCATCGTCTTTGTAGTCAATGAgA AG CAccTTGGC
**TMPRSS2-1083_Rv_Flag_XbaI**	CCAATTTCTAGATTACTTGTCGTCATCGTCTTTGTAGTCCACTAGG TC GTTGAAAGT
**TMPRSS2-948_Rv_Flag_XbaI**	CCAATTTCTAGATTACTTGTCGTCATCGTCTTTGTAGTCTCTCAAA AT CCCCGCAAA
**TMPRSS2-513_Rv_Flag_XbaI**	CCAATTTCTAGATTACTTGTCGTCATCGTCTTTGTAGTCAGGGTGC C AGGACTTCCT
**TMPRSS2-477_Rv_Flag_XbaI**	GGGCCCTCTAGATTACTTGTCGTCATCGTCTTTGTAGTCCTGAAG G ATGAAGTTTGG
**TMPRSS2-444_Rv_Flag_XbaI**	GGGCCCTCTAGATTACTTGTCGTCATCGTCTTTGTAGTCACACCGA T TCTCGTCCTC
**TMPRSS2-411_Rv_Flag_XbaI**	CCAATTTCTAGATTACTTGTCGTCATCGTCTTTGTAGTCTGACACG C CATCACACCA
**TMPRSS2_dAA149-170_Fw**	GGACGAGAATCGGTGTGTGTGCCAAGACGACT
**TMPRSS2_dAA149-170_Rv**	AGTCGTCTTGGCACACACACCGATTCTCGTCC
**TMPRSS2_Rv-YY-FF_P-dead**	CACGGGGGACGGGaAGaACTGAGCCGGATG
**TMPRSS2_Fw-YY-FF_P-dead**	CATCCGGCTCAGTtCTtCCCGTCCCCCGTG
**TMPRSS2_Rv-YY-DD_P-mim**	ACGGGGGACGGGTcGTcCTGAGCCGGATGCAC
**TMPRSS2_Fw-YY-DD_P-mim**	TGCATCCGGCTCAGgACgACCCGTCCCCCG
**TMPRSS2-Y52_Fw-Y-F**	CCCGTGCCCCAGTtCGCCCCGAGGGTCCTG

**TMPRSS2-Y52_Rv-Y-F**	GACCCTCGGGGCGaACTGGGGCACGGGGGA
**6xf-TMPRSS2-Fw_NheI**	CTATAGgctagcGGGCCCACCATGGCTTTGAACTCAGGGTCACCAC CAGCTATTGGACCTTtCTtTGAAAACCATGGA
**6xf-TMPRSS2-Rv**	GACCCTCGGGGCGaACTGGGGCACGGGGGACGGGAAGAACTGA GCCGGATGCACCTCGaAGACAGTGGGGAC
**6xd-TMPRSS2-Fw_NheI**	CTATAGgctagcGGGCCCACCATGGCTTTGAACTCAGGGTCACCAC CAGCTATTGGACCTgACgATGAAAACCATGGA
**6xd-TMPRSS2-Rv**	GACCCTCGGGGCGTcCTGGGGCACGGGGGACGGGTcGTcCTGAG C CGGATGCACCTCGTcGACAGTGGGGAC
**Spike_Fw_NheI_pcDNA3.1**	aagctggctagcatgttcgtgttcct
**Spike-Flag_Rv_XhoI_pcDNA3.1**	ATGCCCctcgagTCActtgtcgtcatcgtctttgtagtctgtatagtGcagtttgacg cccttc
